# Trends in mortality rate in patients with congenital heart disease undergoing noncardiac surgical procedures at children’s hospitals

**DOI:** 10.1038/s41598-021-81161-3

**Published:** 2021-01-15

**Authors:** Viviane G. Nasr, Steven J. Staffa, David Faraoni, James A. DiNardo

**Affiliations:** 1 Department of Anesthesiology, Critical Care and Pain Medicine, Boston Children’s Hospital, Harvard Medical School, 300 Longwood Avenue, Boston, MA 02115 USA; 2 Department of Anesthesia and Pain Medicine, The Hospital for Sick Children, University of Toronto, Toronto, Canada

**Keywords:** Congenital heart defects, Paediatrics

## Abstract

Advances made in pediatric cardiology, cardiac surgery and critical care have significantly improved the survival rate of patients with congenital heart disease (CHD) leading to an increase in children with CHD presenting for noncardiac surgical procedures. This study aims (1) to describe the trend and perioperative mortality rates in patients with CHD undergoing noncardiac surgical procedures at children’s hospitals over the past 5 years and (2) to describe the patient characteristics and the most common type of surgical procedures. The Pediatric Health Information System (PHIS) is an administrative database that contains inpatient, observation, and outpatient surgical data from 52 freestanding children’s hospitals. Thirty-nine of the 52 hospitals submitted data on all types of patient encounters for the duration of the study from 2015 to 2019. The total numbers of non-cardiac surgical encounters among patients with history of a CHD diagnosis significantly increased each year from 38,272 in 2015 to 45,993 in 2019 (P < 0.001). Despite the increase in case numbers, there has been a significant decline in mortality rates to the most recent incidence of 1.06% in 2019. Careful patient selection and medical optimization of patients aligned with specific expertise at dedicated children’s hospitals may lead to improvement in mortality rate. Future studies comparing the outcomes of patients with cardiac disease based on hospital type and volume as well as type of providers may help determine the future of care including potential need for regionalization of noncardiac care for this vulnerable patient population.

## Introduction

The prevalence of moderate to severe congenital heart disease (CHD) in the United States is estimated to be 6 per 1000 live-born full term infants^1^. Advances made in pediatric cardiology, cardiac surgery and critical care have significantly improved the survival rate of patients with CHD leading to increases in the populations of children and adults with significant CHD^2,3^. Children with CHD frequently undergo diagnostic and interventional procedures that require sedation or general anesthesia. Analysis of the Pediatric Health Information System (PHIS) database between 2004 and 2012 demonstrated that 41% of children who had undergone surgery to correct CHD in the first year of life also underwent at least one non-cardiac surgical procedure by age 5^4^. Patients with CHD are generally considered at higher risk for cardiac arrest, perioperative mortality and major morbidity than those without CHD^5–8^.

In a comprehensive analysis of data from the University Hospital Consortium, 191,261 inpatient, non-cardiac procedures occurring between 1993 and 1996 in pediatric patients were analyzed. Mortality was 3.8% in the patients without CHD and 6.0% in the patients with CHD [odds ratio (OR) 3.53, confidence interval (CI) 3.15–3.95]^5^. From 1994 to 2005, the POCA Registry collected data on 373 anesthesia-related cardiac arrests (CA) in children^6^. Thirty-four percent of patients (n = 127) had heart disease (HD) (congenital or acquired). Mortality was 33% in patients with HD as compared to 23% in those without HD^6^. In a recent study, using the American College of Surgeons National Surgical Quality Improvement Program (ACS NSQIP) database, and after exclusion of the patient without CHD and those with minor CHD, the incidence of mortality was 4.7% in a derivation cohort and 4.0% in the validation cohort^9^. These investigations were conducted over a period of two decades and utilized different datasets. In addition, none of those studies have looked at changes in number of cardiac patients with CHD requiring noncardiac procedures over time, neither at the change in mortality rate. Therefore, drawing any inferences about trends in mortality of patients with CHD over time is not possible.

This study aims (1) to describe the trend and perioperative mortality rates in patients with CHD undergoing noncardiac surgical procedures at children’s hospitals over the past 5 years and (2) to describe the patient characteristics and the most common type of surgical procedures performed during that same time interval.

## Methods

### Data source

The Pediatric Health Information System (PHIS) is an administrative database that contains inpatient (more or equal to 24 h stay), observation (< 24 h admission), and ambulatory (outpatient) surgery discharge/encounter data from 52 freestanding children’s hospitals that are part of the Children’s Hospital Association. The PHIS contains International Classification of Diseases 9th and 10th Revision, Clinical Modification (ICD-9-CM) and (ICD-10-CM) diagnostic and procedure codes, date-stamped billing data, and other administrative data^10^. Only hospitals that submitted data from all encounter types during the entire time period of study were included.

### Patient population

Patients less than 18 years of age and undergoing a noncardiac procedure between January 2015 and December 2019 who had an ICD-9-CM diagnosis code for CHD (745.0–747.49) or ICD-10-CM diagnosis code (Q 20.0–Q 26.9) at anytime during the past 10 years 2000 to 2019. Patients who did not have an ICD-9-CM or ICD-10-CM diagnosis of CHD were excluded^11^. Cardiac surgeries and catheter-based interventions were excluded. The frequencies of all noncardiac surgical procedures performed were examined. A non-cardiac surgical procedure was defined as a procedure performed in an operating room (operating room flag in the PHIS database) and with a documented surgical procedure code. The intrinsic surgical risk is the risk of 30-day mortality associated with specific surgical procedures as defined by current procedural terminology (CPT)^12^. Surgical procedures with CPT risk quartiles 1 and 2 comprise the low-risk procedure category, and quartiles 3 and 4 comprise the high-risk procedures.

### Statistical methods

Demographics and patient characteristics are described for encounters of CHD patients undergoing non-cardiac surgery using frequencies and percentages for categorical data, and medians with interquartile ranges (IQR) for continuous data, stratified by year. Mortality rates over time in the entire group and stratified by patient type are expressed as percentages and trends were evaluated using the Cochran–Armitage test across the study years. Trends of the number of cases across the study years were evaluated using generalized linear modeling. The most common cardiac diagnoses based on ICD diagnosis coding is presented by year, as well as the most common non-cardiac procedures with intrinsic surgical risk quartile (ISR). The number of cases based on region of the US is graphically depicted using a heat-map. A two-tailed P value threshold of P < 0.05 was implemented for determining statistical significance. Stata (version 16.0, StataCorp LLC., College Station, TX, USA) was used for all statistical analyses.

## Results

**Figure 1:**
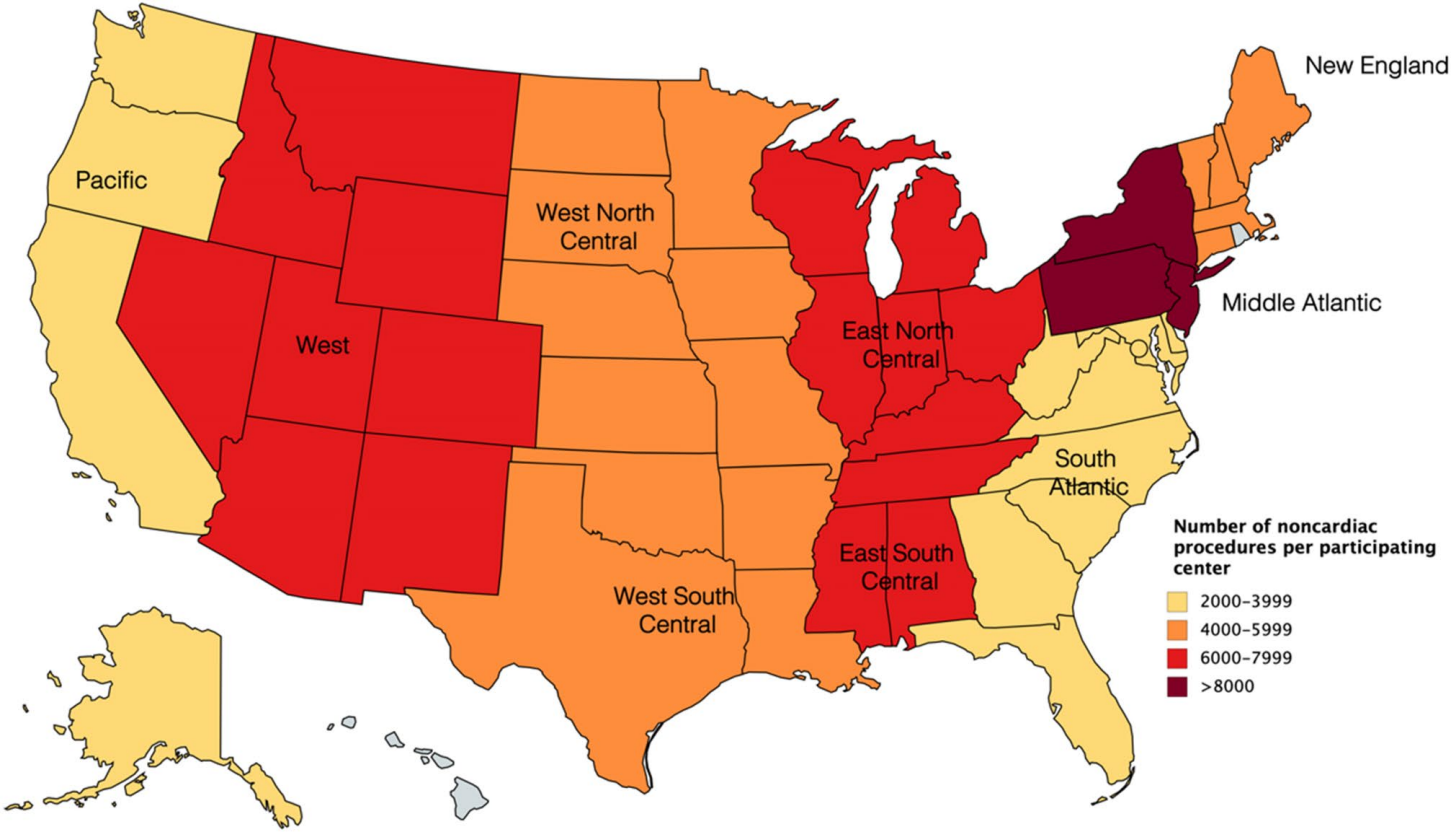
Number of noncardiac procedures per participating center in the United States. The map was created using mapchart.net (https://mapchart.net/usa.html).

Thirty-nine of the 52 participating hospitals in PHIS submitted data on inpatients, outpatients and observation patient types for the duration of the study from 2015 to 2019. For each year from 2015 to 2019, the total numbers of non-cardiac surgical encounters among patients with history of a CHD diagnosis were 38,272, 40,313, 41,454, 43,854, and 45,993, respectively (P < 0.001). Demographics and patient characteristics of patients with CHD presenting for a non-cardiac procedure from 2015 to 2019 are presented in Table 1. The age distribution, race and comorbidities remained similar over the 5-year study period. The distribution of patients in this cohort across the United States is displayed in Fig. 1.

**Table 1:** Demographics and patient characteristics of patients with congenital heart disease undergoing non-cardiac surgery.

Variable	2015	2016	2017	2018	2019
Number of cases	38,272	40,313	41,454	43,854	45,993
**Age (years)**	2.8 (0.9, 6.5)	2.9 (0.9, 6.7)	3 (1, 7)	3.2 (1, 7.2)	3.4 (1.1, 7.6)
Neonates (< 30 days)	3246 (8.5%)	3393 (8.4%)	3459 (8.3%)	3492 (8%)	3520 (7.7%)
Infants (30 days to < 1 year)	6958 (18.2%)	7073 (17.6%)	7082 (17.1%)	7377 (16.8%)	7287 (15.8%)
1 to < 5 years	15,437 (40.3%)	16,092 (39.9%)	16,329 (39.4%)	17,094 (39%)	17,608 (38.3%)
5 to < 12 years	8576 (22.4%)	9399 (23.3%)	10,031 (24.2%)	10,837 (24.7%)	12,092 (26.3%)
12 years or older	4055 (10.6%)	4356 (10.8%)	4553 (11%)	5054 (11.5%)	5486 (11.9%)
**Gender**					
Female	16,179 (42.3%)	17,287 (42.9%)	17,481 (42.2%)	18,626 (42.5%)	19,726 (42.9%)
Male	22,089 (57.7%)	23,017 (57.1%)	23,965 (57.8%)	25,225 (57.5%)	26,263 (57.1%)
Unknown	4 (0.01%)	9 (0.02%)	8 (0.02%)	3 (0.01%)	4 (0.01%)
**Birth weight (kg)**	2594 (1390,3210)	2637 (1580,3210)	2620 (1510,3201)	2630 (1500,3203)	2630 (1535,3218)
**Race**					
White	26,260 (68.6%)	27,775 (68.9%)	28,542 (68.9%)	30,134 (68.7%)	31,178 (67.8%)
Black	6087 (15.9%)	6509 (16.2%)	6816 (16.4%)	7248 (16.5%)	7997 (17.4%)
Asian	1012 (2.6%)	1116 (2.8%)	1254 (3%)	1342 (3.1%)	1411 (3.1%)
Pacific Islander	123 (0.3%)	129 (0.3%)	154 (0.4%)	165 (0.4%)	188 (0.4%)
American Indian	219 (0.6%)	207 (0.5%)	216 (0.5%)	244 (0.6%)	256 (0.6%)
Other	3992 (10.4%)	3783 (9.4%)	3584 (8.7%)	3725 (8.5%)	4088 (8.9%)
**Ethnicity**					
Hispanic or Latino	6152 (16.1%)	6244 (15.5%)	6423 (15.5%)	7110 (16.2%)	7279 (15.8%)
Not Hispanic or Latino	29,918 (78.2%)	31,636 (78.5%)	32,418 (78.2%)	34,366 (78.4%)	36,200 (78.7%)
Unknown	2202 (5.8%)	2433 (6%)	2613 (6.3%)	2378 (5.4%)	2514 (5.5%)
**Insurance**					
Private	15,693 (41%)	16,713 (41.5%)	17,148 (41.4%)	18,089 (41.3%)	18,854 (41%)
Government	21,540 (56.3%)	22,588 (56%)	23,205 (56%)	24,134 (55%)	16,636 (54%)
Other^a^	1039 (2.7%)	1012 (2.5%)	1101 (2.7%)	1631 (3.7%)	10,493 (5%)
**Zip code income**		n = 39,111	n = 40,437	n = 42,801	n = 44,733
1–39,999	16,786 (45.3%)	17,826 (45.6%)	18,596 (46%)	19,694 (46%)	20,317 (45.4%)
40,000–49,999	8792 (23.7%)	9203 (23.5%)	9333 (23.1%)	10,076 (23.5%)	10,505 (23.5%)
50,000–65,999	7733 (20.9%)	8159 (20.9%)	8371 (20.7%)	8755 (20.5%)	9126 (20.4%)
66,000+	3717 (10%)	3923 (10%)	4137 (10.2%)	4276 (10%)	4785 (10.7%)
**Comorbidities**					
Vent dependent	5494 (14.4%)	5837 (14.5%)	6001 (14.5%)	6198 (14.1%)	6175 (13.4%)
Any complex chronic condition	28,389 (74.2%)	28,963 (71.9%)	3009 (72.4%)	32,396 (73.9%)	34,232 (74.4%)
Gastrointestinal	11,274 (29.5%)	12,099 (30%)	12,683 (30.6%)	13,600 (31%)	14,525 (31.6%)
Hematologic or immunologic	1896 (5%)	1910 (4.7%)	1958 (4.7%)	2133 (4.9%)	2207 (4.8%)
Malignancy	1552 (4.1%)	1573 (3.9%)	1615 (3.9%)	1731 (4%)	1884 (4.1%)
Metabolic	1887 (4.9%)	1891 (4.7%)	1982 (4.8%)	2118 (4.8%)	2182 (4.7%)
Neurologic and neuromuscular	5807 (15.2%)	5867 (14.6%)	6170 (14.9%)	6798 (15.5%)	7023 (15.3%)
Renal and urologic	3743 (9.8%)	3852 (9.6%)	3980 (9.6%)	4528 (10.3%)	4740 (10.3%)
Respiratory	6522 (17%)	6749 (16.7%)	7088 (17.1%)	7765 (10.3%)	8030 (17.5%)
Premature and neonatal	3237 (8.5%)	3624 (9%)	4042 (9.8%)	4406 (10.1%)	4538 (9.9%)
Technology dependent	13,670 (35.7%)	14,187 (35.2%)	14,749 (35.6%)	16,005 (36.5%)	16,780 (36.5%)
**Syndromes**					
22q11 deletion syndrome	771 (2%)	763 (1.9%)	789 (1.9%)	842 (1.9%)	937 (2%)
Down syndrome	3889 (10.2%)	4262 (10.6%)	4503 (10.9%)	4596 (10.5%)	4868 (10.6%)
Trisomy 13, 18	204 (0.5%)	213 (0.5%)	243 (0.6%)	258 (0.6%)	291 (0.6%)
**Prematurity (weeks)**					
< 24	480 (1.3%)	504 (1.3%)	467 (1.1%)	489 (1.1%)	499 (1.1%)
24–36	7877 (20.6%)	7987 (19.8%)	8547 (20.6%)	9328 (21.3%)	9888 (21.5%)
37–38	4140 (10.8%)	4668 (11.6%)	4913 (11.9%)	5174 (11.8%)	5861 (12.7%)
**Term (39 weeks or more)**	25,775 (67.4%)	27,154 (67.4%)	27,527 (66.4%)	28,863 (65.8%)	29,745 (64.7%)
**Intensive care unit flag**	33,158 (86.6%)	5333 (13.2%)	5452 (13.2%)	5818 (13.3%)	5859 (12.7%)
**Neonatal intensive care unit flag**	3416 (8.9%)	3665 (9.1%)	3789 (9.1%)	3755 (8.6%)	3821 (8.3%)

Insurance**:** government includes: in-state and out-of-state medicaid, medicare, TRICARE, CHIP and other government.

Intensive care unit (ICU) flag and neonatal intensive care unit (NICU) flag: patient had an ICU or NICU stay during their hospital encounter.

^a^Other payor, self-pay, charity, and unknown.

Table 2 shows the number of encounters and mortality rates over time stratified by patient type. There was a significant increase in the number of inpatient encounters (P = 0.006) and outpatient encounters (P < 0.001) over time. The trend in number of observation unit encounters over time did not reach statistical significance (P = 0.071). There was a significant downward trend in mortality rates in the entire cohort from 2015 to 2019; with mortality rates of 1.16% down to 1.06% (P = 0.01). The median length of stay was similar over the 5 years at 7 days in 2015, 2016, 2017 and 8 days in 2018 and 2019.

**Table 2:** Description of number of encounters, patients, mortality rate and length of stay.

Variable	2015	2016	2017	2018	2019	P value for trend
Number of total encounters	38,272	40,313	41,454	43,854	45,993	< 0.001*
Number of unique patients	28,239	30,102	31,282	32,889	34,744	
Range of number of encounters per patient	1 to 23	1 to 28	1 to 18	1 to 27	1 to 31	
Mortality rate	445/38,272 (1.16%)	524/40,313 (1.30%)	509/41,454 (1.23%)	477/43,854 (1.09%)	489/45,993 (1.06%)	0.01*
**Type of patient**						
Inpatient	14,469	14,961	15,044	15,742	15,839	0.006*
Mortality rate	445 (3.08%)	523 (3.50%)	509 (3.38%)	475 (3.02%)	486 (3.07%)	
Outpatient (ambulatory)	19,719	21,189	22,312	23,911	25,890	< 0.001*
Mortality rate	0 (0%)	0 (0%)	0 (0%)	0 (0%)	2 (0.01%)	
Observation unit	4084	4163	4098	4201	4264	0.071
Mortality rate	0 (0%)	1 (0.02%)	0 (0%)	2 (0.05%)	1 (0.02%)	
**Length of stay**						
Inpatients	7 (2, 27)	7 (3,30)	7 (3,31)	8 (3,30)	8 (3,30)	

Observation means stay strictly less than 24 h.

The number of encounters of patients without a diagnosis of CHD undergoing non-cardiac surgery increased over time (447,503, 457,044, 461,735, 467,146, 489,535; P = 0.014), however the mortality rate in this group did not change significantly across these years (0.13%, 0.13%, 0.11%, 0.12%, and 0.12% respectively; P = 0.226).

Table 3 shows the most common CHD diagnoses for each study year. These include: atrial septal defect, patent ductus arteriosus, ventricular septal defect, congenital insufficiency of aortic valve, stenosis of pulmonary artery, and hypoplastic left heart syndrome. Table 4 summarizes the most common non-cardiac outpatient procedures by year for patients with CHD. These include incision in the middle ear, myringotomy, esophagogastroduodenoscopy, and drainage of left middle ear with open approach. Table 5 summarizes the most common procedures for the encounters admitted for observation, which include: excision and destruction procedures on the pharynx, adenoidectomy, tonsillectomy, endoscopic procedures on the larynx, and bronchoscopy, rigid or flexible. The most common non-cardiac inpatient procedures by year among CHD patients are presented in Table 6. These include: tracheostomy, abdominal wall repair, excision of ileum, restriction of esophagogastric junction, percutaneous endoscopic approach, insertion of feeding device percutaneously and ventricle to peritoneal shunt.

**Table 3:** Most common cardiac diagnoses codes in patients with congenital heart disease presenting for a surgical procedure.

2015		2016		2017		2018		2019	
Diagnosis	(%)	Diagnosis	(%)	Diagnosis	(%)	Diagnosis	(%)	Diagnosis	(%)
Atrial septal defect	38.99	Atrial septal defect	32.28	Atrial septal defect	32.00	Atrial septal defect	31.77	Atrial septal defect	32.47
Patent ductus arteriosus	15.03	Patent ductus arteriosus	14.86	Patent ductus arteriosus	14.75	Patent ductus arteriosus	13.90	Patent ductus arteriosus	13.99
Ventricular septal defect	5.55	Ventricular septal defect	12.54	Ventricular septal defect	11.94	Ventricular septal defect	11.78	Ventricular septal defect	11.49
Congenital insufficiency of aortic valve	4.95	Congenital insufficiency of aortic valve	4.65	Congenital insufficiency of aortic valve	4.75	Congenital insufficiency of aortic valve	5.02	Congenital insufficiency of aortic valve	4.80
Stenosis of pulmonary artery	3.53	Stenosis of pulmonary artery	3.55	Stenosis of pulmonary artery	3.36	Stenosis of pulmonary artery	3.34	Stenosis of pulmonary artery	3.47
Hypoplastic left heart syndrome	3.24	Tetralogy of fallot	2.33	Hypoplastic left heart syndrome	2.25	Hypoplastic left heart syndrome	2.52	Hypoplastic left heart syndrome	2.52

**Table 4:** Most common outpatient noncardiac procedures.

Outpatient procedures	Intrinsic surgical risk
**2015**	
Incision in the middle ear	1
Myringotomy	1
Dentoalveolar procedure	N/A
Endoscopy procedures on the larynx	1
Esophagogastroduodenoscopy	1
**2016**	
Incision in the middle ear	1
Dentoalveolar procedure	N/A
Drainage of left middle ear (open approach)	1
Esophagogastroduodenoscopy	1
Endoscopy procedures on the larynx	1
**2017**	
Incision in the middle ear	1
Dentoalveolar procedure	N/A
Drainage of left middle ear (open approach)	1
Esophagogastroduodenoscopy	1
Endoscopy procedures on the larynx	1
**2018**	
Incision in the middle ear	1
Dentoalveolar procedure	N/A
Esophagogastroduodenoscopy	1
Endoscopy procedures on the larynx	1
Drainage of left middle ear (open approach)	1
**2019**	
Incision in the middle ear	1
Dentoalveolar procedure	N/A
Esophagogastroduodenoscopy	1
Endoscopy procedures on the larynx	1
Excision and destruction procedures on the pharynx, adenoids, and tonsils	1

**Table 5:** Most common procedures as observation (< 24 h admission).

Outpatient procedures	Intrinsic surgical risk
**2015**	
Excision and destruction procedures on the pharynx, adenoids, and tonsils	N/A
Tonsillectomy and adenoidectomy	N/A
Endoscopy- procedures on the larynx	1
Bronchoscopy, rigid or flexible	2–3
Myringotomy	1
**2016**	
Excision and destruction procedures on the pharynx, adenoids, and tonsils	N/A
Tonsillectomy	N/A
Endoscopy-procedures on the larynx	1
Bronchoscopy, rigid or flexible	2–3
Drainage of the middle ear	1
**2017**	
Excision and destruction procedures on the pharynx, adenoids, and tonsils	N/A
Tonsillectomy	N/A
Endoscopy-procedures on the larynx	1
Bronchoscopy, rigid or flexible	2–3
Endoscopic procedure on trachea and bronchi	1
**2018**	
Excision and destruction procedures on the pharynx, adenoids, and tonsils	N/A
Tonsillectomy	N/A
Endoscopy procedures on the larynx	1
Bronchoscopy, rigid or flexible	2–3
Tympanostomy	1
**2019**	
Excision and destruction procedures on the pharynx, adenoids, and tonsils	N/A
Tonsillectomy	N/A
Endoscopy procedures on the larynx	1
Bronchoscopy, rigid or flexible	2–3
Laparoscopic procedures on the stomach	1–2

**Table 6:** Most common inpatient noncardiac procedures.

Inpatient procedures	Intrinsic surgical risk
**2015**	
Gingivoplasty	N/A
Peritoneal adhesiolysis	1,3
Partial resection of the small intestine	3
Restriction of esophagogastric junction, percutaneous endoscopic approach	1
Cerebral ventricle to peritoneal cavity	4
**2016**	
Abdominal wall repair	1–4
Tracheostomy	4
Insertion of feeding device into stomach, percutaneous approach	3
Restriction of esophagogastric junction, percutaneous endoscopic approach	1
Excision of ileum	3–4
**2017**	
Abdominal wall repair	1–4
Tracheostomy	4
Insertion of feeding device into stomach, percutaneous approach	3
Excision of ileum	3–4
Cerebral ventricle to peritoneal cavity	4
**2018**	
Tracheostomy	4
Abdominal wall repair	1–4
Insertion of feeding device into stomach, percutaneous approach	3
Excision of ileum	3–4
Cerebral ventricle to peritoneal cavity, open approach	4
**2019**	
Tracheostomy	4
Abdominal wall repair	1–4
Excision of ileum	3–4
Insertion of feeding device into stomach, percutaneous approach	3
Drainage of cerebral ventricle with device, percutaneous approach	3–4

## Discussion

The number of noncardiac procedures in patients with CHD at PHIS participating children’s hospitals has increased steadily over the past 5 years. Despite the increase in case numbers, there has been a significant decline in mortality rates to the most recent incidence of 1.06% in 2019.

In a recent study using the ACS NSQIP pediatric database, children with CHD undergoing noncardiac surgery had a 30-day mortality incidence of 1.7% in the derivation cohort and 1.5% in the validation cohort (n = 13,129)^13^. This mortality rate is higher than the rate reported in this study. This is most certainly due to differences in the underlying severity of CHD in the two cohorts. In the ACS NSQIP cohort 46.9% of patients had major or severe CHD and 67.9% of patients had unrepaired lesions; in the current cohort > 90% of patients would be classified as having minor CHD. Previous work by our group has demonstrated that in children with CHD, patient comorbidities, and severity of the cardiac lesion are the predominant predictors of 30-day mortality^13^. These differences in patient demographics as regards CHD severity are likely due to the fact that while the PHIS data includes inpatient, outpatient and observation unit encounters at dedicated children’s hospitals, the ACS NSQIP data includes only inpatient pediatric cases and the participating hospitals include freestanding hospitals, children’s hospitals within larger hospitals, specialty children’s hospitals or general acute care hospitals with a pediatric wing^10–14^.

While the mortality rate in CHD patients improved, the mortality rate in patients without CHD remained relatively constant at 0.12% over the 5-year study period. The mortality rate in this cohort is comparable to that seen in other studies. Among the 183,423 children included in the 2012 to 2014 ACS NSQIP database, the incidence of mortality was 0.5% (563/115,229) in the derivation cohort and 0.4% (290/67,904) in the validation cohort^15^. Among the 13,530 cases included in a subsequent external validation cohort, the incidence of 30-day mortality was 0.21% (29/13,530)^16^. In a more recent study using the ACS NSQIP database, the overall 30-day mortality in 367,065 surgical cases encompassing 659 unique Current Procedural Terminology codes was 0.34%^13^. Obviously, these studies speak to the high quality of perioperative care currently rendered to children undergoing surgery.

The most common types of noncardiac procedures performed on CHD patients remained constant over the 5-year study period. Gastrointestinal and otolaryngologic procedures were the most common. This is consistent with a previous study which demonstrated that general surgical and otolaryngologic procedures to be the most common procedures performed in the first 5 years of life in children requiring infant cardiac surgery^4^. Assignment of our intrinsic surgical risk (ISR) classification to the procedures performed on patients in this cohort demonstrated that most of the noncardiac procedures performed in the outpatient and observation settings are low risk with an ISR of 1^13^. A limitation of the ISR is that tonsillectomies are not included in the classification as this procedure is not reported to the ACS-NSQIP database from which the ISR was derived. The ISR for procedures performed on the inpatient cohort ranged from 1 to 4. Clearly, the more complex surgical procedures are performed in the inpatient setting as would be expected.

Careful patient selection and medical optimization of patients aligned with specific expertise at dedicated children’s hospitals may lead to additional improvement in mortality rate over time in patients with CHD undergoing non-cardiac surgery. It has been demonstrated that collaboration and transparency between institutions leads to reductions in-hospital mortality, duration of mechanical ventilation, length of stay and major complications following pediatric cardiac surgery. In children’s hospitals participating in the Pediatric Cardiac Critical Care Consortium (PC4) outcomes improved over a 2-year period while non-participating hospitals showed no improvement in outcomes over this time interval^17^. It has been suggested that regionalization of care at specialized hospitals can reduce mortality for congenital heart surgery as well^18^. Children with CHD, especially those with unrepaired lesions or with a residual lesion burden and compromised cardiovascular status require an individualized approach to anesthetic and surgical care delivered by trained multidisciplinary teams^19,20^. Practitioners including cardiologists, intensivists, and primary care physicians who care for children with CHD must address a number of challenges including identifying the best location for procedures (e.g., children’s hospital), identifying qualified team (e.g., cardiac anesthesiologists, surgeons), and utilizing the expertise of the non-cardiac subspecialties (e.g., nephrology, hematology, pediatricians).

This study has several limitations. The PHIS database is an administrative database and miscoding of CHD may occur^21–23^. Since the results are based on only the 39 children’s hospitals that participated during the past 5 years by contributing all types of patients encounters, the generalizability of the results is limited. While using ICD 9 and 10 in the PHIS database provides the information about the cardiac diagnosis and surgical procedure, it lacks the granularity in regard to the severity of the cardiac disease which is necessary to further categorize the cardiac disease based on functional severity and degree of residual cardiac lesions. In addition, we looked at the trends over 5 years, which led to the inclusion of ICD 9 in the 2015 data. The ICD 9 coding were converted to ICD 10 in 2015. We used the conversion of ICD 9 to ICD 10 to sum the number of procedures and cardiac diagnosis.

In conclusion, this analysis of the PHIS database points out that the mortality rate of patients with CHD undergoing noncardiac procedures is improving despite the increase in number of noncardiac procedures performed per year. Future studies comparing the outcomes of patients with cardiac disease based on hospital type and volume as well as the provider experience (pediatric cardiac versus general pediatric anesthesiologist) may help determine the future of care including potential need for regionalization of noncardiac care for this vulnerable patient population.
